# The Assessment of CD56 and CD117 Expressions at the Time of the Diagnosis in Multiple Myeloma Patients

**DOI:** 10.4274/tjh.2016.0394

**Published:** 2017-08-02

**Authors:** Funda Ceran, Mesude Falay, Simten Dağdaş, Gülsüm Özet

**Affiliations:** 1 Ankara Numune Training and Research Hospital, Clinic of Hematology, Ankara, Turkey

**Keywords:** CD56, CD117, Flow cytometry, Multiple myeloma

## Abstract

**Objective:**

The purpose of this study is to investigate the relationship between the CD56 and CD117 expressions and the clinical and laboratory findings in multiple myeloma (MM) patients.

**Materials and Methods:**

Analyses of multiparametric flow cytometry data obtained from the diagnostic bone marrow aspirations of a total of 34 newly diagnosed MM patients were assessed retrospectively. CD56 and CD117 expressions of the patients were compared with their stages and clinical parameters. The staging was performed according to the International Staging System (ISS).

**Results:**

Of the patients, 58.8% had ISS stage 1-2 MM while 41.2% had stage 3 MM. The number of CD56-positive patients was 29, whereas the number of CD117-positive patients was 13. There was no statistical difference between the CD56 and CD117 expressions and extramedullary involvement and lytic bone lesions. The median beta-2 microglobulin level was higher in the CD117-negative group (p=0.047). CD56 and CD117 expression levels were found to be lower in advanced-stage patients than in early-stage ones (p=0.026 and p=0.017). The lactate dehydrogenase (LDH) levels were high in advanced-stage patients, and an inverse relationship was found between LDH level and CD117 expression.

**Conclusion:**

Our findings that the CD56 and CD117 expression levels are lower in advanced stages than earlier stages and that LDH level and CD117 expression have an inverse relationship in patients with newly diagnosed MM suggest that CD56 and CD117 expressions may be prognostic markers for MM.

## Abstract (tr)

**Amaç:**

Bu çalışmanın amacı multipl miyeloma (MM) hastalarında CD56 ve CD117 ekspresyonları ile klinik ve laboratuvar bulguları arasındaki ilişkiyi araştırmaktır.

**Gereç ve Yöntemler:**

Yeni tanı toplam 34 MM hastasının kemik iliği aspirasyon örneklerinde çalışılan multiparametrik flow sitometri analizleri retrospektif olarak değerlendirildi. Hastaların CD56 ve CD117 ekspresyonları hastalık evreleri ve klinik parametreleri ile karşılaştırıldı. Evreleme, Uluslararası Evreleme Sistemi (ISS)’ne göre gerçekleştirildi.

**Bulgular:**

Hastaların %58,8’i ISS evre 1-2 MM iken, %41,2’si evre 3 MM’ydi. CD56 pozitif hasta sayısı 29, CD117 pozitif hasta sayısı 13’tü. CD56 ve CD117 ekspresyonları ile ekstramedüller tutulum ve litik kemik lezyonları arasında istatistiksel fark yoktu. Ortalama beta-2 mikroglobulin düzeyi, CD117 negatif grupta daha yüksekti (p=0,047). İleri evre hastalarda CD56 ve CD117 ekspresyon düzeyleri erken evre hastalara kıyasla daha düşük bulundu (p=0,026 ve p=0,017). İleri evre hastalarda laktat dehidrogenaz (LDH) düzeyleri yüksekti ve LDH düzeyi ile CD117 ekspresyonu arasında ters bir ilişki saptandı.

**Sonuç:**

Yeni tanı MM hastalarında ileri evrelerde CD56 ve CD117 ekspresyon düzeylerinin erken evre olanlardan daha düşük olması ve LDH düzeyi ile CD117 ekspresyonunun bulduğumuz ters ilişkisi, CD56 ve CD117 ekspresyonlarının MM için prognostik belirteç olabileceğini düşündürmektedir.

## INTRODUCTION

Multiple myeloma (MM) is a clonal hematologic malignancy that occurs as a result of the accumulation of malignant plasma cells in the bone marrow. Apart from the fact that conventional morphologic examination is essential in the diagnosis of MM and the assessment of response to therapy, the importance of multiparametric flow cytometry (MFC) in MM is growing. Its limited use in MM can be attributed to several factors such as sampling differences, the loss of plasma cells during analysis, adherence to the tubes, and the use of initial aspiratory samples in morphological assessment [1,2,3]. MFC is a valuable tool in distinguishing malignant plasma cell populations from the reactive/normal ones in samples. Aberrant immunophenotyping is assistive in the identification of these populations and available in most myeloma cases. It can also be used in the follow-up of minimal residual diseases (MRDs) [4,5]. MFC is a rapid, sensitive, and reliable method in the identification of clonality and aberrant antigenic expressions. It is possible to analyze both surface and intracytoplasmic antigens simultaneously, and it depicts quantitative results. It is better than immunohistochemical examination [1,2]. The European Myeloma Network reports the required antibodies in the panel as CD38, CD138, CD19, CD45, CD56, CD20, CD117, CD28, and CD27 and recommends that gating, which is of the utmost importance, be carried out according to CD38, CD138, and/or CD45 antibodies. CD19 is negative, CD56 is positive or negative, and CD38 is positive in malignant plasma cells [1,4,6]. Among the aberrant antigenic expressions, there may be antigens such as CD56, CD117, CD19, CD27, CD28, and CD33 [1,2,3,4,5,6,7]. In recent years, some of them have gained increasing attention with regard to their effects as adhesion molecules and their relations with the microenvironment. They are also becoming specific targets for curative treatments. There are studies that assess the relationship between MM prognosis and immunophenotype [8,9,10,11,12]. In our study, the relationship between the CD56 and CD117 aberrant expressions in malignant plasma cells in MM patients at the time of diagnosis and clinical and laboratory parameters were retrospectively assessed.

## MATERIALS AND METHODS

A total of 34 MM patients, whose bone marrow aspiration samples were analyzed by MFC immunophenotyping at the time of diagnosis, were assessed retrospectively. The conventional cytogenetic analyses for del 13q, del 17p, and immunoglobulin H (IgH) translocations with fluorescence in situ hybridization were assessed. Written consent and local ethics committee approval were received.

Ethylenediaminetetraacetic acid tubes were used for the bone marrow samples whereas polystyrene tubes were used for the test samples. Beckman Coulter (BC) (Brea, CA, USA) phosphate-buffered saline OptiLyse solution was utilized in order to wash the cells and to keep the erythrocytes away. The monoclonal antibodies (MoAb), which were used in marking, were obtained from BC. CD45 phycoerythrin-cyanine 5 (PC5), CD19 phycoerythrin-Texas red (ECD), CD38 phycoerythrin (PE), CD138 fluorescein isothiocyanate (FITC), CD10 PE, CD20 PC5, CD117 ECD, CD56 PE, cytoplasmic kappa FITC, and cytoplasmic lambda PE MoAb were used and four-color analysis was applied. Bone marrow samples (100 µL) were put into polystyrene tubes and 20 µL of MoAb was added. Upon 20 min of incubation in the dark, the samples were washed. Isotopic control was applied for each analysis. Acquisition was done in the FC500 BC machine with at least 50,000 cell counts. CD38+ and CD138+ cells were gated and analyzed in the CXP program. Antigen expressions were accepted as positive when they were >20%.

### Statistical Analysis

Statistical analysis was performed with SPSS 20 software. Normal data distributions were assessed with the Shapiro-Wilk test. The comparison of two groups of numeric variables was made with the t-test and Mann-Whitney U test. The chi-square test and Fisher’s exact test were used in the comparison of categorical data. The relationship among numerical variables was investigated with Pearson and Spearman correlation analyses. Stepwise multivariate logistic regression analysis was used in the identification of the independent predictors that could affect the numeric CD56 negativity, CD117 negativity, and stage 3 risks.

## RESULTS

**Figure 1:**
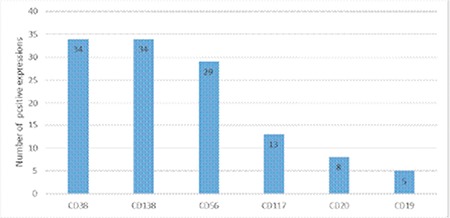
The number of positive antigen expressions in patients with multiple myeloma (n=34).

The clinical features and the demographic data of the patients at the time of diagnosis (Table 1) and overall antigen expressions (Figure 1) are provided below.

**Figure 2:**
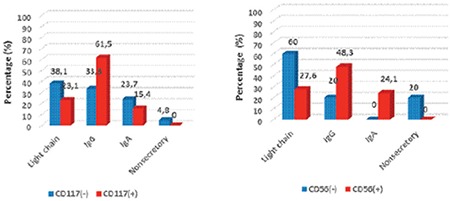
Multiple myeloma subtypes according to CD56 and CD117 expressions.

The plasma cell rate assessed morphologically in the bone marrow aspiration samples was higher than that of the samples used for MFC. The rate of the patients with stage 1-2 MM was 58.8% and the rate of those with stage 3 MM was 42.2%. Plasmacytoma was present in 14.7% of the patients. The distribution of the MM subtypes according to CD56 and CD117 expressions are shown in Figure 2. CD56-negative patients had a lower average age than those with positive CD56 (50.2±14.1 and 62±10.3 years, p=0.0032). CD56-positive patients were more often at stage 1-2 (65.5%), whereas negative ones were more often at stage 3 (80%).

**Figure 3:**
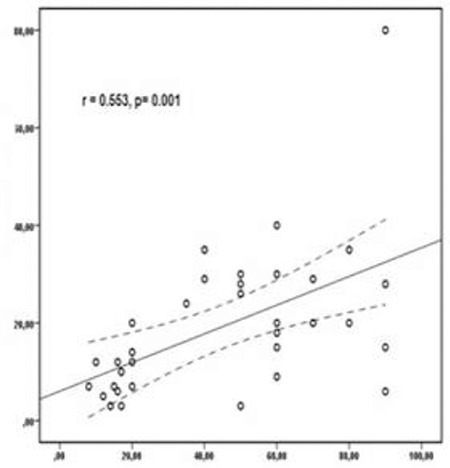
The plasma cells (%) in multiparametric flow cytometry and the plasma cells (%) in the bone marrow aspiration.

While the rate of the patients with light-chain immunoglobulin type was higher in the CD56-negative group (60% vs. 27.6%), the IgG and A types were higher in the CD56-positive group (respectively 20% vs. 48.3%, 0% vs. 24.1%, p=0.027), and there was no difference between kappa and lambda light-chain types (p=0.732). No relationship was found between the existence of plasmacytoma and CD56 expression, which may derive from the small number of patients (p=0.717). The beta-2 microglobulin (B2M), C-reactive protein (CRP), creatinine, and sedimentation levels were higher in the CD56-negative group when compared with the positive group. The plasma cell rate of the CD56-negative group was higher in MFC; the rate of lytic lesions was lower than in the positive group, but that was not statistically significant. The rate of patients with stage 1-2 MM was 76.9% in the CD117-positive group whereas the rate of those with stage 3 MM was 23.1%; however, this was not statistically significant (p=0.153). Though there was not a statistically significant difference among the immunoglobulin subtypes (p=0.271), the light-chain type was seen more often in the CD117-negative group while the IgG and A types were seen more often in the CD117-positive group. There was no difference between kappa and lambda light-chain types. The median B2M level was significantly higher in the CD117-negative group than the positive one at 6.3 vs. 3.5, respectively (p=0.047). The plasma cell rates identified in MFC were not different (p=0.365). Though the lytic lesion rates were higher in the CD117-negative group (47.6% vs. 23%), no statistical difference was observed (p=0.286). The distribution of the demographic and clinical findings of the patients according to CD56 and CD117 expressions are shown in Table 2. Stages 1 and 2 MM were more common in double-positive patients, whereas B2M was lower (p=0.01); the platelet number was higher (p=0.039) and the creatinine level was lower (p=0.041). There were three double-negative patients, and the bone marrow plasma cell rate for all of them was ≥50%. Two of them were at stage 3. IgH translocation was found in one patient [t (11;14)]. The light-chain type was more common in advanced-stage MM. The median lactate dehydrogenase (LDH) level was higher (p=0.037). CD56 and CD117 expression levels were lower in advanced-stage MM than in early-stage MM (p=0.026 and p=0.017, respectively). A negative correlation was observed among CD117 and both hemoglobin (p=0.023, r=-0.389) and LDH levels (p=0.048, r=-0.381). A positive correlation was found between the plasma cell percentage in MFC and both the plasma cell percentage in bone marrow aspiration (Figure 3) and CRP (respectively p=0.001, r=0.553; p=0.007, r=0.452), whereas a negative one was detected between the plasma cell percentage in MFC and the platelet number (p=0.048, r=-0.341).

Cytogenetic analysis was performed for 21 patients. There was del 13q in 2 patients, del 17p in one patient, and IgH heavy-chain mutation in one patient [t (11; 14)]. The patient with del 17p was CD117-negative. One of the patients who were detected with del 13q was CD117-positive while the other one was CD117-negative. The patient with t(11,14) was both CD56- and CD117-negative. However, statistical analysis could not be conducted due to the small number of patients. Six of the assessed patients were lost (17.64%). Three of these patients were CD117-negative.

## DISCUSSION

The features of the antigenic profiles of malignant cells are beneficial in both the diagnosis and the identification of the prognostic markers in different hematologic diseases. During the last decade considerable evolvement of MFC has led to studies that report some aberrant expressions of different markers in most of the plasma cell malignancies. The expression of these markers is typically used in the discrimination of malignant plasma cells from benign ones. The immunophenotyping of plasma cells in MM does not differ whether the patient received therapy or not, but an immunophenotypic shift can be seen in those that were treated with targeted therapy [13]. Current risk stratification systems in MM mainly depend on the cytogenetic/molecular findings, but they do not include any parameter concerning aberrant antigenic expressions. MFC is more sensitive than immunohistochemical analyses in identifying aberrant antigenic expressions. The main applications of MFC in MM are assessment of MM progression from monoclonal gammopathy of undetermined significance (MGUS), follow-up of MRDs, and detection of the prognostic markers and identification of new treatment goals for MM [1,2,3,6,7,8,12,14,15,16,17,18,19,20,21,22,23].

CD56 is a neural cell adhesion molecule (NCAM) related to the axon growth in normal embryogenesis. It is expressed in most of the malignant plasma cells (about 70%-80%) and its deficiency may be related to an aggressive disease [9,10,21,23]. CD117 is an essential hematopoietic growth factor receptor with tyrosine kinase activity. It cannot be expressed by normal plasma cells. It is observed to be positive in approximately 33% of MM patients [8,24,25,26]. In this study, the CD56 and CD117 expressions in 34 newly diagnosed MM patients were retrospectively assessed. The light-chain type was found to be more common in the CD56-negative patients, whereas the IgG and IgA types of MM were more in the CD56-positive group. Pan et al. [26] found similar results recently, but there was no difference between CD56-positive and -negative patients with regard to kappa and lambda light-chain types. Similarly, Van Camp et al. [27] showed that the lambda light-chain type was more common in CD56-negative patients. Previously, Sahara et al. [28] showed that thrombocytopenia, renal failure, and increase in B2M level were more common in CD56-negative MM cases. We found that creatinine and B2M levels were higher and platelet number was lower in the CD56-negative group (respectively p=0.125, p=0.158, and p=0.368), but these differences did not reach statistical significance.

The relationship between CD56 expression and the existence of lytic lesions was assessed. Although there were fewer lytic lesions in the CD56-negative group (p=0.682), there was no statistical difference between these groups. CD56 expression is considered to have a role in lytic lesion generation by leading to a decrease in osteoblast functions. Osteoblasts also express CD56. Thus, the NCAM-NCAM interactions between the plasma cells and stromal and osteoblastic cells result in a decrease in bone matrix production [10,20,29]. Lytic lesions can be seen at lower rates despite the fact that there are more bone marrow plasma cell infiltrations. Extramedullary involvements are more common in CD56-negative patients due to the fact that CD56 is a marker related to the fixation of the plasma cells to the stromal structure, and an inverse relationship was found between CD56 expression and the plasma cells in circulation [28]. The rate of the existence of extramedullary involvement in our study was parallel to literature reports; nevertheless, no relationship was observed between this rate and CD56 expression (p=0.717). If the sample size were larger, a more meaningful result could be obtained.

CD117 expression has been shown to be decreased during the progression from MGUS to the advanced stage of MM [8,24]. CD117 positivity is considered to have a relationship with good prognosis [8,11,12,26,30]. In a recent study, Pan et al. [26] found a relationship between CD117 positivity and longer overall survival. They also detected that overall survival has a relationship with CD56, stage, and B2M.

In accordance with the literature [8,12], B2M level was found to be lower in the CD117-positive group (p=0.047). As an increased B2M level is accepted as a key indicator for advanced stage, this finding showed a relationship between CD117 negativity and advanced stage. In agreement with previous reports [12,31], CD117 expression was found to be lower in advanced-stage MM (p=0.017). Shin et al. [31] showed a relationship between advanced-stage MM and CD117 negativity. In our study, an inverse relationship was observed between CD117 expression levels and LDH levels (p=0.048, r=-0.381). The decrease in CD117 expression may support the increase in LDH levels in advanced stages. This could suggest disease progression and may be a sign of poor prognosis. The CD56 expression level was found to be lower in advanced-stage MM, like CD117 expression levels (p=0.026). In previous studies, during the progression from MGUS to symptomatic myeloma and approaching advanced-stage MM, CD56 and CD117 expression levels were shown to be decreased [7,8]. This can be explained as a result of features of CD56 and CD117, which might act as adhesion and anchor molecules. These features might be effective in the homing of malignant plasma cells and the limiting of the disease. This may result from the association of these markers with the microenvironment. Therefore, low expression levels can lead to the spreading of plasma cells, progression of the disease, advanced stages, and poor prognosis [26]. We think that our study reflects this progression.

Prognosis studies in MM include cytogenetic analyses. In particular, CD117 negativity is in relation with the high-risk karyotype and IgH heavy-chain mutations. In our study, CD117 was also negative in a patient with del 17p and one in two patients with del 13q among the patients for whom cytogenetic analyses were performed. In the patient with IgH mutation, both CD56 and CD117 were negative. Pozdnyakova et al. [32] revealed a relationship between poor cytogenetic features and CD56 and CD117 negativity in their studies. CD117 negativity was observed especially more often with poor cytogenetic features. They suggested that more information about the cytogenetic analyses can be obtained when the expressions of these two markers are assessed in patients for whom cytogenetic analyses cannot be applied. In the study of Mateo et al., [12] a relationship was shown between CD117 negativity and both IgH translocations and del 13q.

## CONCLUSION

There were several limitations of this study. The major limitation was that it was a single-center retrospective study and the patient population was relatively small. Since it was a retrospective study, there were some missing cytogenetic data in the patient records.

In conclusion, the lower levels of CD56 and CD117 expressions in advanced-stage disease and also the inverse relationship between LDH level and CD117 expression may support the importance of these expressions as prognostic markers in MM. We think other MoAb that show different aberrant antigenic expressions in addition to CD56 and CD117 should be added to MFC panels and further studies are required to evaluate cytogenetic features together.

## Figures and Tables

**Table 1:**
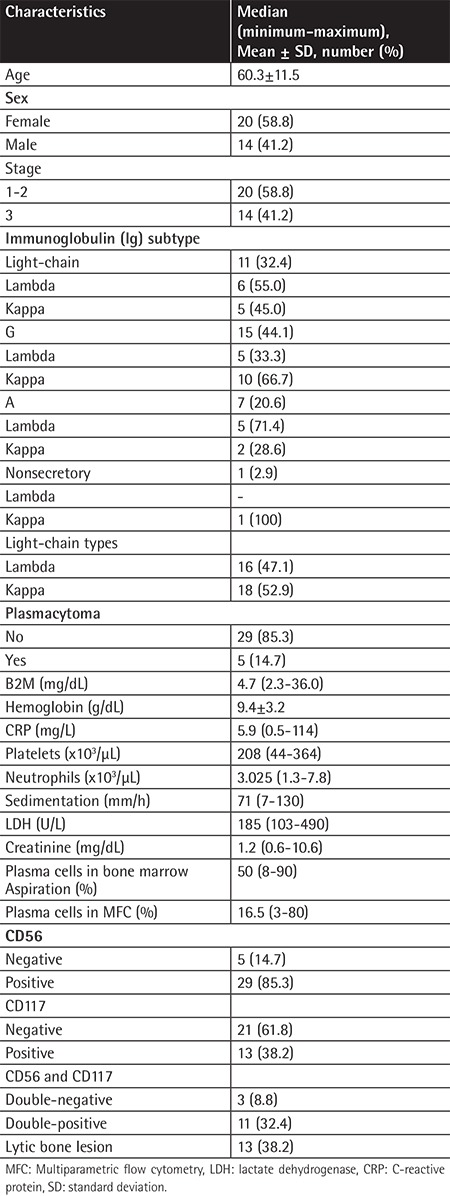
The clinical characteristics of the patients (n=34).

**Table 2:**
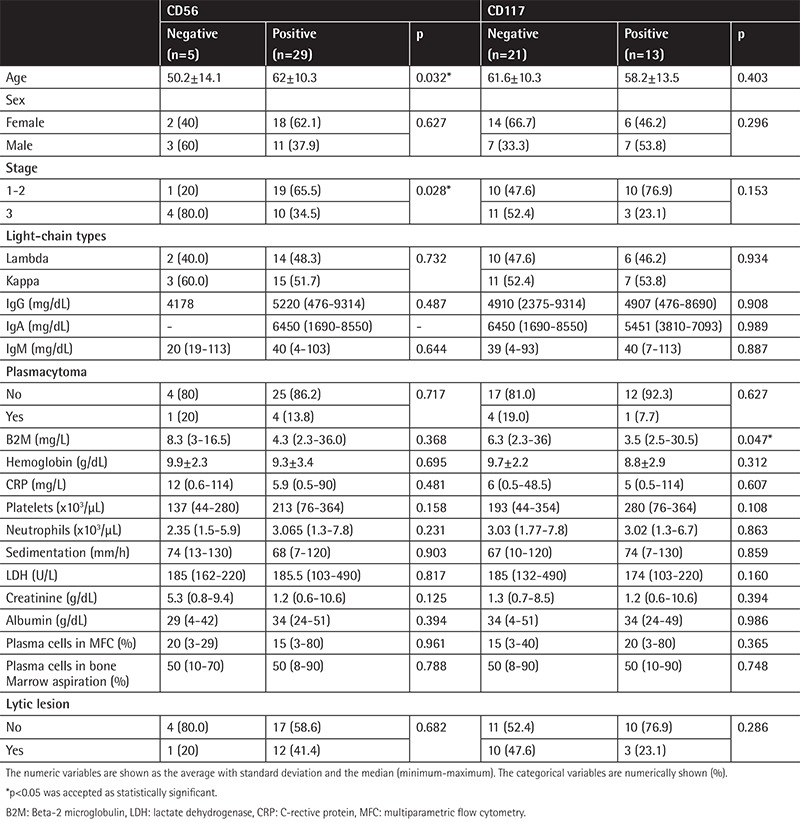
The distribution of the demographic and clinical findings of the patients according to CD56 and CD117 expressions.
